# Dynamic Navigated Computer-Guided Incision and Corticotomy: Description of a Novel Technique and a Case Report

**DOI:** 10.1155/crid/4867363

**Published:** 2025-11-20

**Authors:** Davide Brilli, Matteo Giansanti, Francesca Germanò, Isabella Cauli, Michele Cassetta

**Affiliations:** Department of Oral and Maxillofacial Sciences, Sapienza University of Rome, Rome, Italy

**Keywords:** case report, computer-guided surgery, corticotomy, dynamic computer-guided surgery, navigation, piezocision

## Abstract

This report describes an innovative dynamic computer-guided piezocision technique that integrates preoperative digital planning with real-time intraoperative navigation to accelerate orthodontic treatment while minimizing complications. A 24-year-old female patient who requested short-term orthodontic treatment underwent a minimally invasive, flapless corticotomy using a dynamic system. Preoperative optical scanning and cone-beam computed tomography were performed for comprehensive three-dimensional surgical planning. Intraoperatively, reference trackers were fixed and calibration was performed, enabling precise guidance. A conventional scalpel, adapted with a drill-tag, was employed to execute fully guided soft tissue incisions, and a piezoelectric cutting insert was calibrated for corticotomy cuts. The dynamic navigation system provided continuous real-time feedback on instrument position during the surgical procedure, guiding the surgeon regarding location, depth, and angulation, while ensuring optimal irrigation to reduce thermal risks. The fully guided dynamic piezocision technique enabled the execution of corticotomies following the planned cuts without surgical complications such as damage to dental roots or periodontal tissues. The dynamic approach allowed intraoperative adjustments and improved irrigation, reducing the risk of thermal injury. Fully guided dynamic piezocision may enhance surgical accuracy and safety compared to traditional methods by combining preoperative digital planning with real-time dynamic navigation.

## 1. Introduction

In recent years, the main purposes of the orthodontic research field and treatments are no longer only to carry out a treatment aimed at improving esthetics and dental-facial functions but also to reduce the duration of treatment and further reduce the risks of complications associated with long-term treatments (such as decreasing patient compliance, caries, and external root resorption). For these reasons, a topic of great concern in orthodontics is the acceleration of orthodontic tooth movement [1].

A wide spectrum of procedures and techniques has been described in the literature during the last six decades, such as traditional corticotomy and piezocision, microosteoperforations, low-frequency vibrations, photobiomodulation low-intensity pulsed ultrasound, periodontally accelerated osteogenic orthodontics, and pharmacological approaches [2]. Although there is a considerable number of techniques described, corticotomy has been described as one of the most effective and safe procedures in orthodontic tooth movement acceleration [3]. Some authors reported that the corticotomy procedure shortens the conventional orthodontic time since teeth can be moved two to three times faster [3, 4].

The regional acceleratory phenomenon (RAP) is considered the biological basis of accelerated tooth movement [4]. The RAP can be stated as a physiological tissue reaction to a noxious stimulus (including the system of orthodontic forces), that, in the alveolar bone, leads to an increased activity of osteoclastic cells, activation of metabolic processes, and increased bone turnover.

These cellular and molecular processes lead to transient osteopenia and increased bone remodeling [4]. In the alveolar bone, the RAP occurs typically in the healing process of the alveolar sockets after tooth extraction, in periodontal disease, after surgery and trauma and during orthodontic tooth movement [5].

In 1959, Köle outlined one of the first traditional corticotomy techniques that required the incision and elevation of wide mucoperiosteal flaps. However, due to the aggressive nature and postoperative discomfort, this method is no longer executed [6].

More recently, to ensure a safe, flapless and minimally invasive approach, as well as a predictable and customizable surgical procedure, computer-guided surgery was also introduced into piezoelectric corticotomy procedures.

Two main approaches have been highlighted in the field of computer-guided piezoelectric corticotomy: static, employing surgical templates, or dynamic/navigated computer-guided surgery systems.

Navigation systems allow for a guided approach with real-time feedback on the relative location of the piezoelectric cutting tip and the patient's jaw. These dynamic systems were initially introduced in implantology [7, 8] but are also being used for the insertion of temporary anchorage devices (TADs) [9] and piezoelectric corticotomy [10]. Fujinaka et al. [10] recently described an innovative piezocision procedure through computer-guided navigation; however, the technique described lacks a totally guided workflow throughout the intervention by the dynamic system and also does not involve the incision of the soft tissues through the use of the guidance offered by the navigation system.

This report is aimed at describing and establishing a novel piezocision technique using dynamic computer-guided navigation technology, that we called “Navicision,” based on preoperative planning to allow, during the surgical phase, a real-time fully guided approach.

## 2. Case Presentation

The clinical investigation was conducted at the Department of Oral and Maxillo-Facial Surgery of Sapienza University of Rome. The clinical investigation was conducted following the CARE guidelines and ethical principles of the Declaration of Helsinki. The subject was informed of the content, risks, and benefits of the study, and written consent was obtained. The investigation was independently reviewed and approved by the local ethics committee (No. 7782). Written consent was obtained for the execution of intraoral photographs.

### 2.1. Diagnosis and Etiology

**Figure 1:**
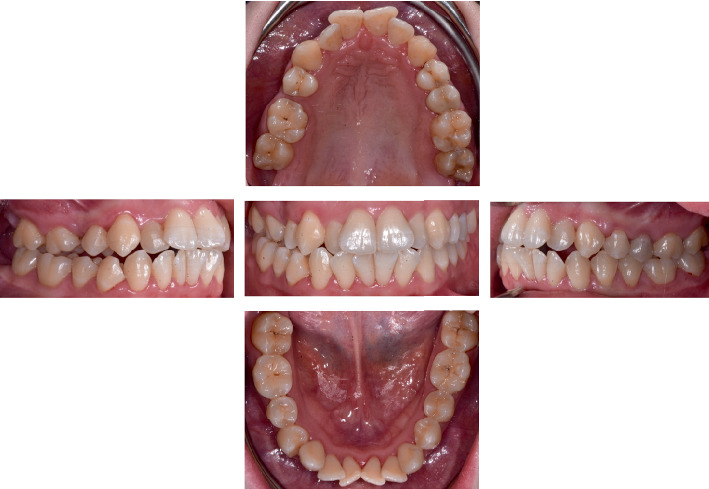
Pretreatment intraoral photographs.

The patient was a 24-year-old female, who requested a short-term orthodontic treatment. The extraoral and intraoral examination showed a symmetric face, competent lips, straight profile, and short face. Furthermore, a right Class II and left Class I molar relationship, crowding, reduced overjet, and overbite were detected (Figure 1). The clinical examination has not found any periodontal or temporomandibular joint pathologies.

**Figure 2:**
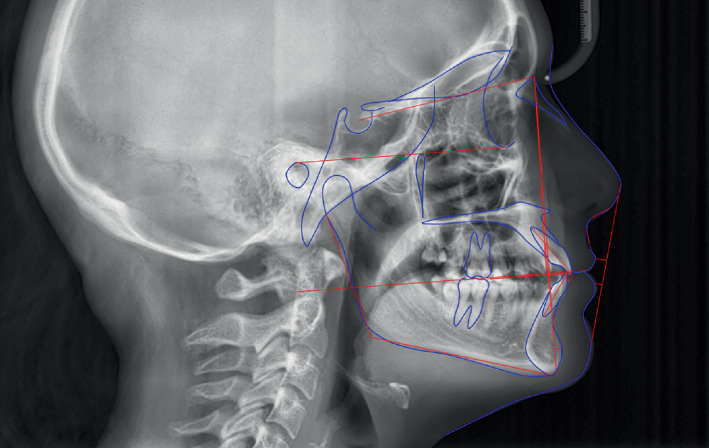
Pretreatment cephalometric record.

The cephalometric analysis demonstrated a skeletal Class I pattern (ANB, 1.93°; wits appraisal, 1.32 mm), with a normal anteroposterior position of the maxilla (SNA, 82.39°) and mandible (SNB, 80.47°), a hypodivergent pattern (FMA, 18.66°), a reduced interincisal angle (interincisal angle, 116.85°) with a lower incisal proclination (IMPA, 107.29°). (Figure 2 and Table 1).

### 2.2. Treatment Objectives

The primary objectives of the treatment were to accelerate orthodontic tooth movement and reduce the overall duration of orthodontic therapy. Specifically, the approach aimed to:

• Enhance the efficiency of tooth movement by harnessing the RAP, thereby shortening treatment time.
• Ensure a safe, minimally invasive procedure that protects dental roots and periodontal structures from iatrogenic damage.
• Integrate preoperative digital planning with real-time dynamic navigation to optimize the precision of both soft and hard tissue incisions and to mitigate potential thermal injuries during corticotomy.

### 2.3. Treatment Alternatives

Some alternative therapeutic strategies were considered:

- Conventional orthodontic treatment without adjunctive surgical intervention
- Static computer-guided piezocision, utilizing a prefabricated surgical template, and orthodontic treatment
- No treatment

These Objectives and Alternatives

The treatment plans were proposed and explained to the patient. The objectives and alternatives were carefully considered to provide an optimized balance between treatment efficacy, patient comfort, and procedural safety. The decision was to perform a dynamic computer-assisted corticotomy and sequentially fixed multibracket orthodontic therapy using indirect bonding.

### 2.4. Treatment Progress

To decrease the duration of the orthodontic treatment, a dynamic navigated-computer-guided corticotomy, a novel technique, was planned and performed.

To ensure a safe and minimally invasive surgical procedure, the corticotomies were carried out using the Navident computer-guided dynamic navigation system (ClaroNav Technology Inc., Toronto, Canada).

The subject underwent optical scanning and cone-beam computed tomography (CBCT) examination of the jaws; STL (Standard Triangulation Language) and DICOM (Digital Imaging and Communications in Medicine) files were superimposed to determine appropriate sites.

CBCT images were taken using the Scanora 3Dx cone-beam device (Soredex, Tuusula, Finland). The exposure parameters were as follows: 10 mA, 90 kV, 10 mA, total scanning time of 15.0 s, effective radiation time of 4.0 s, and voxel size of 0.2 mm, and the field of view was limited to the upper and lower jaws. The STL files were collected using the Medit i700 intraoral scanner (Seoul, South Korea).

**Figure 3:**
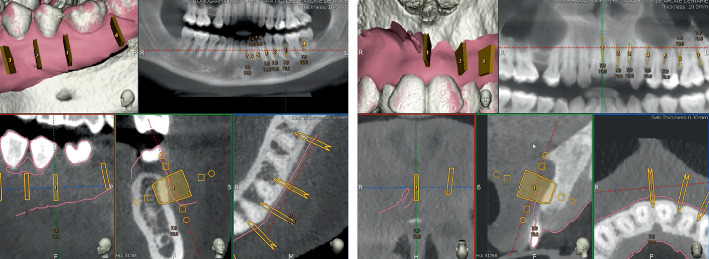
Planning of the corticotomy cuts through the software Navident and the dedicated “saw-cut” tool.

Once the files were superimposed, the Navident software (ClaroNav Technology Inc., Toronto, Canada), through the specific and dedicated “Add Saw-Cut” tool, was employed to plan corticotomies in terms of direction, length, width, and depth. This tool allows planning the three-dimensional position of the piezoelectric cutting tip and, further, permits choosing the dimension of the piezoelectric cutting insert simulating the surgical field and guiding the position of the microsaw with real-time feedback during the surgery (Figure 3).

The space between the roots of each tooth was assessed, and a longitudinal axis, running parallel to each root, was established. Surgical cuts were executed at a distance of at least 2 mm from the papilla, penetrating through the thickness of the cortical layer entirely. These cuts were designed on the basis of the use of the OT7A piezoelectric cutting insert (Mectron, Carasco, Genoa, Italy), considering a 0.75 mm dimension of the microsaw.

The surgical phase began by temporarily placing two reference pattern tags—called head tracker and jaw tracker—on the subject. Subsequently, the patient was asked to rinse with 0.2% chlorhexidine mouthwash for 1 min preoperatively. Plexus anesthesia was performed using mepivacaine with adrenaline 1:100.000 (Molteni Farmaceutici, Scandicci, Florence, Italy).

**Figure 4:**
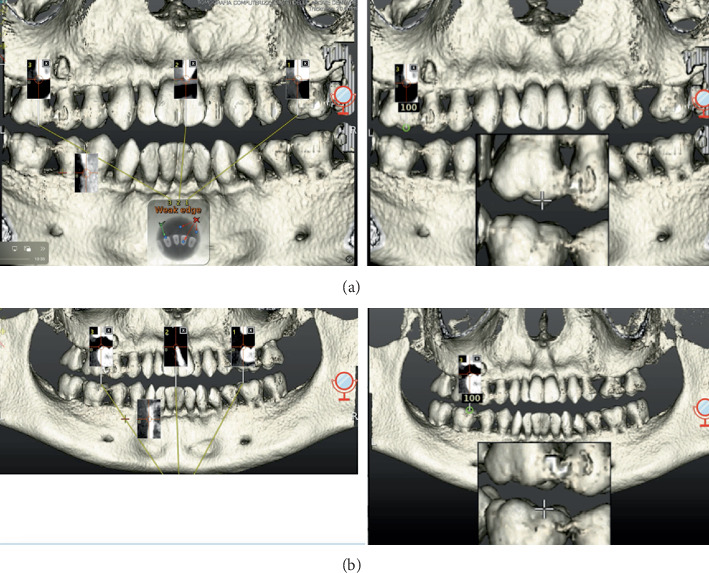
(a) Trace registration for upper arch. (b) Trace registration for lower arch.

The calibration phase was then carried out, selecting three points of reference on the three-dimensional reconstruction of DICOM files produced by the Navident software (ClaroNav Technology Inc., Toronto, Canada) for each jaw, and, through the “tracer” instrument, these points were detected in the dental arches. This procedure permitted matching the relative position of the jaws to the virtual planning (Figure 4).

**Figure 5:**
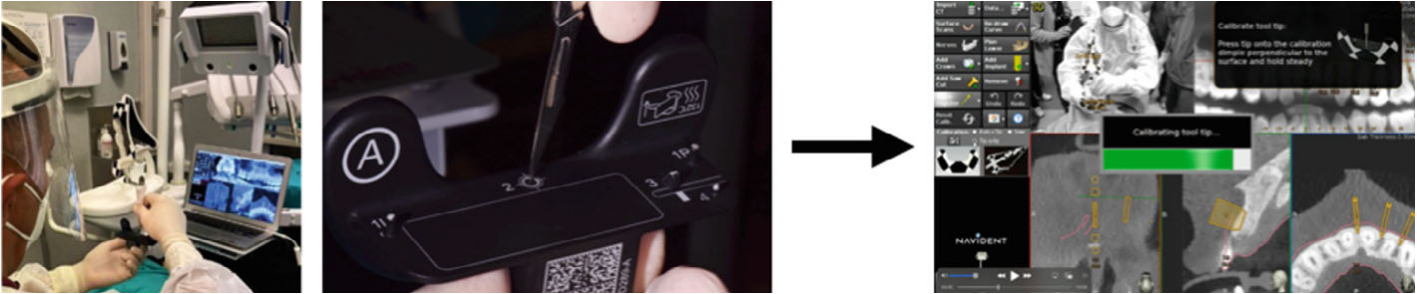
Illustration of the “trace registration” process of the scalpel blade. The operator holds the “calibrator” while in the other hand holds the scalpel, on which has been placed the insert called “drill-tag.” At this point, the operator holds the tip of the scalpel on the surface of the “calibrator” so that the software is able to recognize the shape and size of the scalpel blade.

**Figure 6:**
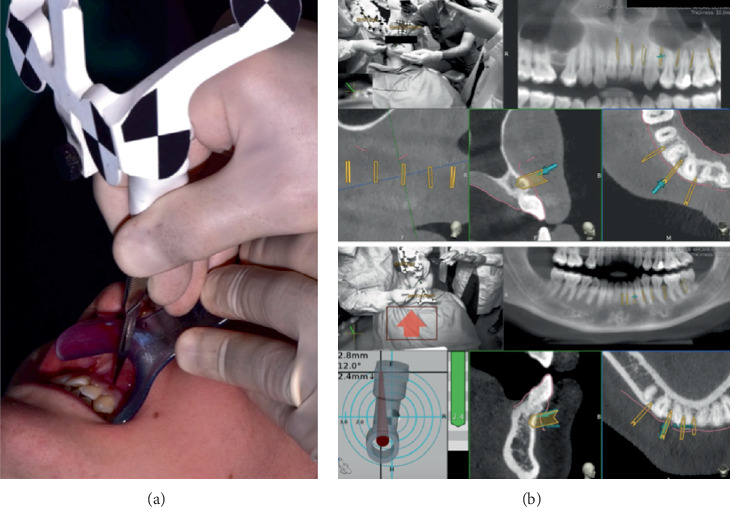
(a) Intraoperative photograph during guided soft tissue incision. (b) Real-time view produced by the software, in which the operator can see the real-time position of the surgical scalpel tip (blue arrow) over the patient's arches and the programmed cuts (orange signs).

A fully guided vertical full-thickness gingival incision, below the interdental papilla, crossing the periosteum and reaching the alveolar bone surface, was then performed using a Number 11 scalpel blade by using the dynamic system. This technique first involved adapting the “drill-tag” to the scalpel handle to render the surgical instrument detectable by the dynamic navigation system. Following this, the instrument registration was carried out using the “calibrator” instrument to provide the system with information regarding the shape and length of the scalpel blade (Figure 5). A calibration accuracy check was performed prior to the surgical phase, ensuring acceptable deviation values of ≤ 0.5 mm. The scalpel blade was then positioned at the preplanned soft tissue incision site, and the incision was entirely guided by the software in terms of position, depth, and angle (Figure 6).

**Figure 7:**
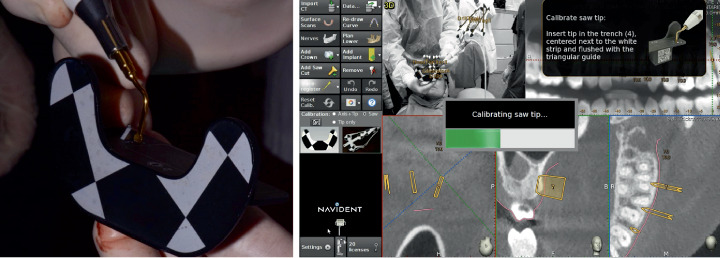
Illustration of the “trace registration” process of the microsaw. The operator holds in one hand the “calibrator,” while in the other hand holds the piezoelectric handpiece, on which has been placed the insert called “drill-tag.” Then, the operator holds the tip of the microsaw on the surface of the “calibrator” so that the software can recognize the shape and size.

Thereafter, the microsaw used was calibrated, through the “calibrator” tool, to give the Navident software information about the insert size. A calibration accuracy check was also executed in this phase, considering acceptable deviation values ≤ 0.5 mm (Figure 7).

Corticotomies were performed using the Mectron Piezosurgery White (Mectron, Carasco, GE, Italy) piezoelectric surgical device and OT7A piezoelectric cutting insert (Mectron, Carasco, GE, Italy).

**Figure 8:**
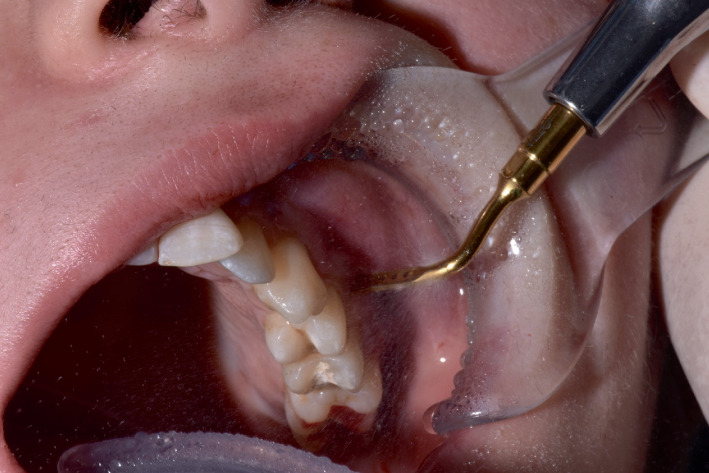
Intraoperative clinical photo during the execution of the corticotomy cut. It is possible to note that, given the absence of a surgical guide, the irrigation flow is directed onto the tissues, allowing a more efficient cooling of the surgical site.

**Figure 9:**
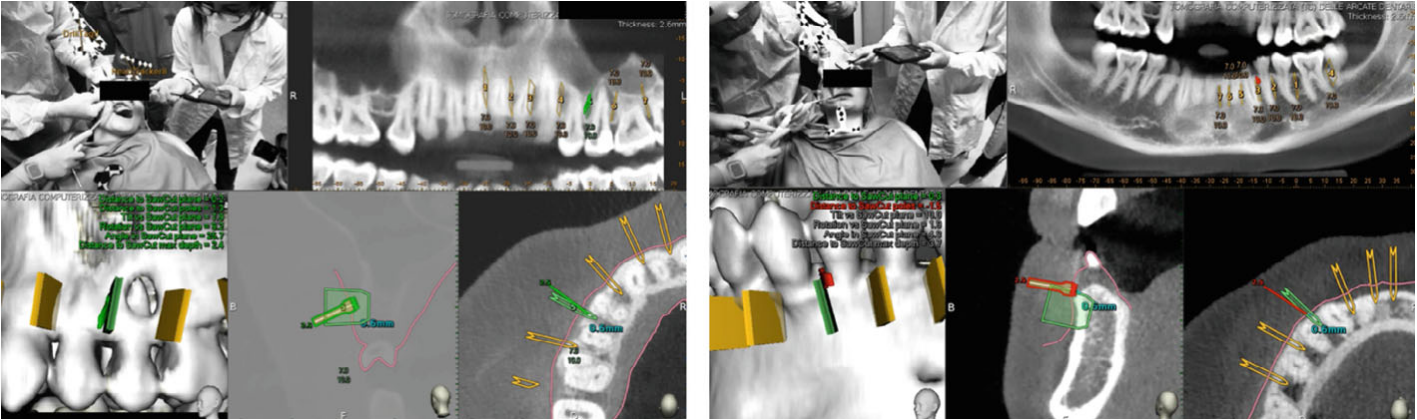
Navigation view during the execution of fully guided corticotomy cuts. The software identifies the position of the handpiece and microsaw in relation to the arches in real time, indicating the axis and the ideal depth programmed in two-dimensional and three-dimensional views.

**Figure 10:**
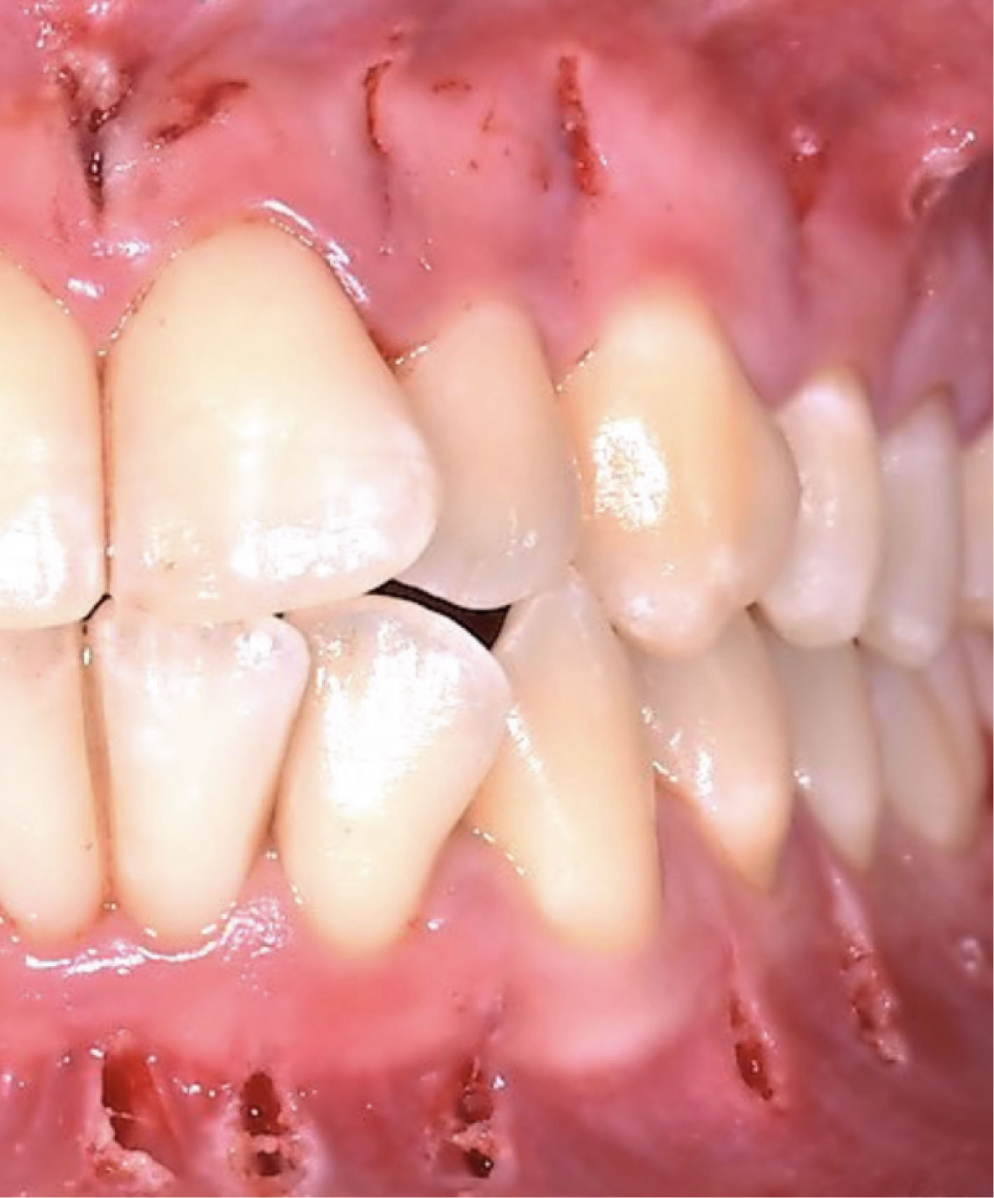
Intraoral photograph at the end of the surgical phase. It is possible to note the absence of signs of severe overheating on soft tissues.

The fully guided corticotomy cuts were achieved through the gingival incisions, extended across the entire thickness of the cortical layer, and real-time indications and quantitative tridimensional feedback of the relative position of the microsaw were indicated by the software, following the virtual planning made in the planning phase (Figures 8, 9, and 10).

At the end of the surgery, the patient underwent a CBCT examination to confirm the absence of damage to the anatomical structures.

**Figure 11:**
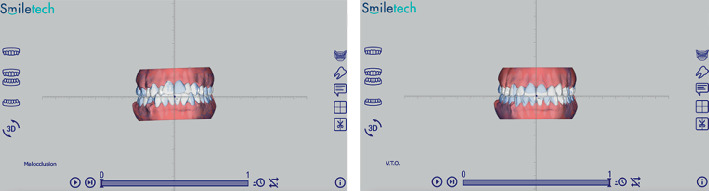
Preview and simulation of orthodontic movement via the Smiletech viewer.

**Figure 12:**
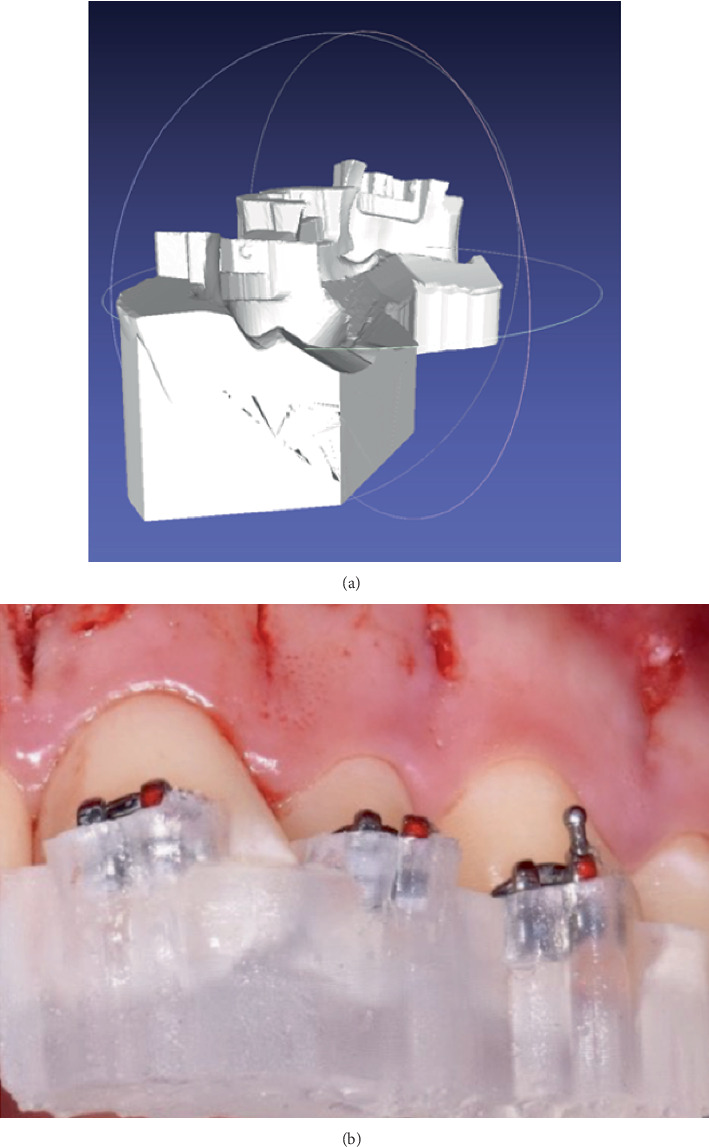
(a) Partial design of the CAD/CAM device for indirect bonding. (b) Indirect orthodontic bonding through the use of the CAD/CAM device.

**Figure 13:**
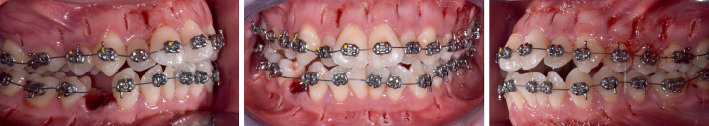
Posttreatment intraoral photographs at the end of the surgical and orthodontic phase.

The orthodontic treatment started the same day, at the end of the surgical phase, using the indirect orthodontic bonding technique to achieve more accurate bracket positioning while minimizing undesired dental movements. Before the surgical phase, a digital planning process was carried out by superimposing DICOM and STL files using the I/D module of the OnyxCeph^3^ software (Chemnitz, Germany) and the Smiletech viewer (Ortodontica Italia srl, Rome, Italy). This process allowed for the simulation of orthodontic movements and the production of indirect bonding CAD/CAM guides for both the upper and lower arches, utilizing the AccuFab-L4K 3D printer (Shining 3D Dental, Zhejiang, China) and the surgical guide SG01 photopolymer resin (Shining 3D Dental, Zhejiang, China). Subsequently, following the surgical phase, the CAD/CAM guides for indirect bonding were positioned, and the brackets were then placed accordingly. The orthodontic appliance activation was performed concurrently on the same day (Figures 11, 12, and 13).

At the end of the procedure, the patient received the graphic rating scale for pain (GRS), a visual analog scale in which a numerical scale (from 0 to 10) is added with the endpoints defining extreme limits such as “no pain at all” and “pain as bad as it could be.” The patient was asked to mark her pain level on the line between the two endpoints daily for the first seven postoperative days, after the clinician gave verbal instructions to assess pain and on pain management as the painkillers had been forbidden. Only in case of severe pain was the patient instructed to contact the clinician for a painkiller prescription, but this did not happen.

## 3. Results

The fully guided, minimally invasive, and flapless, dynamic computer-guided piezocision technique allowed the execution of corticotomies with no surgical complications, such as root or marginal periodontium damage, and did not require hard or soft tissue grafting. Further, no unexpected events were detected.

The absence of a surgical template allowed for obtaining good irrigation, avoiding the onset of thermal injuries to soft and hard tissues.

The highest pain intensity was observed on day 1, with a score of 6/10 on the GRS; on day 2, the pain decreased to 3/10; on day 3, it further declined to 1/10, and no pain was reported on subsequent days.

The total duration of the surgical procedure was 1 h and 10 min.

## 4. Discussion

Both static and dynamic computer-guided corticotomy techniques ensure minimally invasive corticotomies without flap reflection and also permit avoiding damage to anatomical structures such as dental roots or marginal periodontium.

Several benefits could be attributed to the navigated computer-guided corticotomy system compared to the static system. First of all, the use of stereolithographic surgical templates in static techniques, even in computer-aided implantology, obstructs the correct irrigation of the piezoelectric cutting insert causing overheating of the tissues; to overcome this issue, some authors indicated that it is necessary to reduce the pressure on the insert and do back-and-forth movements of the insert from the surgical guide in order to allow the outflow of bone fragments and cooling [11]. Conversely, navigated computer-guided surgery systems do not use surgical guides, ensuring the correct irrigation and thus reducing thermal injuries to soft tissues [10], and also allow reducing the production time and costs of the surgical template.

Further, some authors stated several other advantages of dynamic systems, such as the better visualization of the surgical field [10, 12] and the possibility of making intraoperative changes and adjustments [10].

However, dynamic navigation systems also present significant limitations. One of the most relevant is the high initial investment cost, which can be a barrier to adoption, particularly in smaller practices with limited case volumes. Moreover, the learning curve for dynamic navigation can be steep, as previously reported [13–15], requiring both dedicated training sessions and a period of clinical practice before proficiency is achieved. In fact, according to these studies, optimal efficiency and precision are typically achieved only after several completed cases. By contrast, in static computer-guided surgery, Cassetta et al. demonstrated the absence of a learning curve [16]. These considerations mean that the choice between static and dynamic systems should take into account not only technical benefits but also economic feasibility and the time investment required for skill acquisition.

Fujinaka et al. [10] recently described an innovative piezocision procedure through computer-guided navigation, with results comparable to the present study. However, the novel technique established by this study had two substantial differences. First, the execution of preoperative digital planning; in fact, this technique not only permitted constant feedback of the relative position of the microsaw and jaws such as the technique proposed by Fujinaka et al. [10], but the software also indicated the location, angulation, and penetration depth of the piezoelectric cutting insert compared to the planned cuts accurately, allowing it to constantly guide the microsaw in the planned position. Second, the other difference is the scalpel guidance in order to perform a computer-guided soft tissue incision. In fact, it should be emphasized that a correct soft tissue incision is an essential condition for the proper performance of piezocision; that is, the soft tissue incision must coincide with the cortical incision. Since the soft tissue incision is preparatory to the hard tissue incision, the two tracts must coincide, and this condition can only be met if both incisions are guided.

Furthermore, to the best of the author's knowledge, this is the first study that demonstrates not only the guidance of the microsaw by the dynamic navigation system following preoperative planning but also the feasibility of the use in dynamic navigation of conventional surgical instruments, such as scalpel blades. This allows for guided soft tissue incisions, making it beneficial not only for performing corticotomies but also more broadly in any field of oral and maxillofacial surgery.

## 5. Conclusions

The dynamic Navicision technique may offer a reproducible approach for guided corticotomies, combining digital planning and real-time navigation to enhance surgical accuracy, supporting the clinician in avoiding damage to adjacent anatomical structures through feedback of the relative position of the scalpel blade and microsaw, and minimizing soft tissue trauma. Further studies are needed to validate its effectiveness and reproducibility.

## Figures and Tables

**Table 1:** Pretreatment cephalometric analysis.

**Parameters**	**Measures**	**Mean values**
SNA	82.39°	82 ± 2°
SNB	80.47°	80 ± 2°
ANB	1.93°	2 ± 2°
Wits appraisal	1.32 mm	0 ± 2 mm
Go-Gn to SN	24.81°	32 ± 3°
FMA	18.66°	25 ± 4°
IMPA	107.29°	90 ± 5°
FMIA	54.05°	65 ± 5°
U1 to maxillary plane angle	120.40°	109 ± 4°
Interincisal angle	116.85°	131 ± 5°

## Data Availability

The data that support the findings of this study are available from the corresponding author upon reasonable request.
